# S5-2 Co-creating a feasible and valid physical literacy assessment tool for children for use in both educational and community physical activity settings, within England

**DOI:** 10.1093/eurpub/ckad133.024

**Published:** 2023-09-11

**Authors:** Jade Morris

**Affiliations:** University of Bradford, UK

## Abstract

**Purpose:**

Physical literacy (PL) has been described as a gateway to lifelong participation in physical activity, with relevance to the education, health, and sports contexts. That said, it remains a contested term in research and practice, meaning existing PL assessment tools (PLAT) are inconsistent, with divergent ways to assess PL and the encompassing domains. Furthermore, PLATs often lack feasibility, especially when looking to gain population-level data. Therefore, we are developing a feasible and valid PLAT for children (ages 5-11) for use in both educational and community physical activity settings, within England.

**Method:**

To develop an England-specific PLAT, the project includes four stages: (1) a review of reviews to identify existing strengths and weaknesses of existing tools, (2) identifying stakeholders’ facilitators and barriers to using a PLAT in an education and/or community sports setting, (3) co-developing a feasible PLAT through a multi-stakeholder co-creation event (researchers, practitioners, policymakers), and (4) pilot testing to assess the tools validity and feasibility.

**Results:**

The results of the scoping review of reviews revealed very few tools exist that assess all domains of PL. Of the identified tool, where the validity and reliability of the tool were more promising, the feasibility of the tool lacked. These findings were akin to tools assessing one domain of PL. Stages 2 and 3 are currently in process. We anticipate sharing findings from the stakeholder survey, and insights from the co-creation event that will have led to a newly developed PLAT for England.

**Conclusion:**

The co-created PLAT tool will bring together stakeholders across the education, sport, and health sector to gain consensus around the importance of assessing children’s PL in a bid to improve their physical activity levels. We anticipate the co-developed tool will provide surveillance data on children’s PL levels, which will enhance our understanding of the specific needs of children and young people to improve their PL. Thus, better targeting and tailoring support for children and young people to be physically active throughout their lives.

**Support/Funding Source:**

The project has been externally funded by Sport England and the London Marathon Group.

